# Supplementary material for: Maternal Health Care Service Utilization in the Post-Conflict Democratic Republic of Congo: An Analysis of Health Inequalities over Time
Source: Healthcare (Basel). 2023 Oct 31;11(21):2871. doi: 10.3390/healthcare11212871 (PMC10649172; doi:10.3390/healthcare11212871)
Supplement: Supplementary file 1 [file healthcare-11-02871-s001.zip › Additional file S1 STROBE checklist_Maternal Health Care Services Utilization in the post-conflict DRC.pdf]

Additional file S1 STROBE checklist_Maternal Health Care Services Utilization in the post-conflict Democratic Republic of Congo: Analysis of health inequalities over time

	Item No	Recommendation	Page No
**Title and abstract**	1	(*a*) Indicate the study's design with a commonly used term in the title or the abstract	This is a cross-sectional study design of the demographic and health survey (2007 & 2013-2014) that was covered in the title and abstract (Page 1).
		(*b*) Provide in the abstract an informative and balanced summary of what was done and what was found	The abstract summarizes the background, methods, results, and conclusions of the study (Pages 1-3).
**Introduction**			
Background/rationale	2	Explain the scientific background and rationale for the investigation being reported	The scientific rationale for this study has been explained in the four paragraphs of the background section (Pages 3-5)
Objectives	3	State-specific objectives, including any prespecified hypotheses	The objectives can be found in the last paragraph of the background section (Page 5)
**Methods**			
Study design	4	Present key elements of study design early in the paper	The key elements of the study design are presented in the methodology section.<br>First wave: a total of 9,002 households were randomly selected, with a household response rate of 99.3%; 9,995 women aged 15-49 were interviewed.<br>Second wave: a total household sample of 18,360 was randomly selected, with a household response rate of 98.6 %; 18,827 women aged 15-49 were interviewed (Page 6)
Setting	5	Describe the setting, locations, and relevant dates, including periods of recruitment, exposure, follow-up, and data collection	The Demographic and Health Surveys were conducted in the DRC to collect data from a nationally representative sample of households. The survey employed a standardized, and rigorous sampling and data collection methodology. They are described in the first sub-section of the methods. Two data sets were analyzed.<br>Interviews were carried out by trained interviewers using standardized questionnaires.<br>(Pages 6-7)
Participants	6	(*a*) *Cohort study*—Give the eligibility criteria and the sources	The selection of participants for qualitative data collection is

		and methods of selection of participants. Describe methods of follow-up<br>*Case-control study*—Give the eligibility criteria and the sources and methods of case ascertainment and control selection. Give the rationale for the choice of cases and controls<br>*Cross-sectional study*—Give the eligibility criteria and the sources and methods of selection of participants	described in the Sources of Data, in the methods section.<br>The subjects of this study were restricted to mothers aged 15-49 years who:<br>-were asked whether they had at least three visits for ANC checkups, received at least one TT injection, or underwent the following checkups at least once during antenatal visits – weight, height, blood pressure, blood test, urine test –and whether they received information regarding pregnancy for the last birth during the five years preceding the survey<br>-were asked where their children were born, who assisted during the deliveries, and many other characteristics of delivery.<br>-had their last birth were asked "if they did have any check-ups within 48 hours after delivery?" and whether or not the "women underwent any health check-up by a health professional after delivery. (Pages 7-8)
		(*b*) *Cohort study*—For matched studies, give matching criteria and number of exposed and unexposed<br>*Case-control study*—For matched studies, give matching criteria and the number of controls per case	N/A
Variables	7	Clearly define all outcomes, exposures, predictors, potential confounders, and effect modifiers. Give diagnostic criteria, if applicable	Detailed definitions of all outcomes and exposure variables were provided. The outcome of interest included ANC, delivery, and PNC.<br>All key variables of the study are described in the selection of variables sub-section of the methods.<br>(Pages 7-8-9)
Data sources/ measurement	8*	For each variable of interest, give sources of data and details of methods of assessment (measurement). Describe the comparability of assessment methods if there is more than one group	Data sources have been described in the first and second sub-section of the Methods, while measurements /assessments are discussed under Statistical data analysis of the methods (Pages 5 – 12)
Bias	9	Describe any efforts to address potential sources of bias	To avoid potential sources of bias, a standardized data collection instrument was used, and women were interviewed confidentially and anonymously. We have described the

			data quality and methodology robustness in the methods.<br>(Pages 6-7)
Study size	10	Explain how the study size was arrived at	A multi-stage cluster survey design was used to represent the population of the DRC. Survey clusters were selected to be representative of all provinces. Within clusters, households were randomly selected proportional to the population size, and within each household, women ages 15–49 years were consented and interviewed.
Quantitative variables	11	Explain how quantitative variables were handled in the analyses. If applicable, describe which groupings were chosen and why	Quantitative variables were reported as odds ratio (OR) and relative risk ratio (RRR). Explained in the Methods subsections (Pages 9-12).
Statistical methods	12	(*a*) Describe all statistical methods, including those used to control for confounding	Described in the Statistical data analysis sub-section of the Methods.<br>(Pages 9-12)
		(*b*) Describe any methods used to examine subgroups and interactions	N/A
		(*c*) Explain how missing data were addressed	N/A
		(*d*) *Cohort study*—If applicable, explain how loss to follow-up was addressed<br>*Case-control study*—If applicable, explain how matching of cases and controls was addressed<br>*Cross-sectional study*—If applicable, describe analytical methods taking account of the sampling strategy	Sampling weights available in the DHS databases were applied to the performed statistical calculations.
		(*e*) Describe any sensitivity analyses	N/A
**Results**			
Participants	13*	(a) Report numbers of individuals at each stage of study—e.g. numbers potentially eligible, examined for eligibility, confirmed eligible, included in the study, completing follow-up, and analysed	N/A
		(b) Give reasons for non-participation at each stage	N/A

		(c) Consider use of a flow diagram	N/A
Descriptive data	14*	(a) Give characteristics of study participants (e.g. demographic, clinical, social) and information on exposures and potential confounders	Characteristics of the study participants have been described in the results sub-section (Page 9).<br>Table 1. presents descriptive characteristics of all respondents in the DRC by survey year (2007 & 2013-2014)
		(b) Indicate number of participants with missing data for each variable of interest	N/A
		(c) *Cohort study*—Summarise follow-up time (e.g., average and total amount)	N/A
Outcome data	15*	*Cohort study*—Report numbers of outcome events or summary measures over time	N/A
		Case-control study—Report numbers in each exposure category, or summary measures of exposure	N/A
		Cross-sectional study—Report numbers of outcome events or summary measures	All selected Maternal healthcare services variables are described in Table 6.
Main results	16	(*a*) Give unadjusted estimates and, if applicable, confounder-adjusted estimates and their precision (e.g., 95% confidence interval). Make clear which confounders were adjusted for and why they were included	Presented in Tables 2, 3, 4 & 5, and Figure 1 and throughout the results section by thematic topics (Magnitude of inequality (RQ1), Inequality distribution (RQ2), and Inequality trends (RQ3)<br>(Pages 12-18)
		(*b*) Report category boundaries when continuous variables were categorized	N/A
		(*c*) If relevant, consider translating estimates of relative risk into absolute risk for a meaningful time period	N/A
Other analyses	17	Report other analyses done—e.g. analyses of subgroups and interactions, and sensitivity analyses	N/A
**Discussion**			
Key results	18	Summarise key results with reference to study objectives	Summarized in the first sub-section of the Discussion as follows:<br>While continuous improvements in the utilization of MHCS were found at different stages of pregnancy, several aspects remain inequitable. Our study found important variations in the utilization of MHCS by geographic region, socioeconomic households, and survey years (Page 19)
Limitations	19	Discuss limitations of the study, taking into account sources of potential bias or imprecision. Discuss both direction and magnitude of any potential bias	Discussed in the Study strengths and Limitations sub-section of the Discussion. (Pages 22-23)

Interpretation	20	Give a cautious overall interpretation of results considering objectives, limitations, multiplicity of analyses, results from similar studies, and other relevant evidence	Interpreted in the sub-section of the strengths and limitations (Page 22).
Generalisability	21	Discuss the generalisability (external validity) of the study results	Findings assume that there has been a declining trend for some variables from 2007 to 2013-2014.<br>Several factors could explain this, such as place of residence, ethnicity, education level, religious affiliation, wealth index, and year of the survey were associated with inequality in the utilization of ANC, delivery care, and PNC.
**Other information**			
Funding	22	Give the source of funding and the role of the funders for the present study and, if applicable, for the original study on which the present article is based	Explained under the declarations sub-section that No funding was obtained for this study.<br>(Page 25)

*Give information separately for cases and controls in case-control studies and, if applicable, for exposed and unexposed groups in the cohort and cross-sectional studies.

Note: An Explanation and Elaboration article discusses each checklist item and gives methodological background and published examples of transparent reporting. The STROBE checklist is best used in conjunction with the explanatory article, which is available freely on the Web sites of PLoS Medicine (http://www.plosmedicine.org/), Annals of Internal Medicine (http://www.annals.org/), and Epidemiology (http://www.epidem.com/).
Information on the STROBE Initiative is available at www.strobe-statement.org. (all accessed on 28 February 2020).
