# Supplementary material for: Maternal Health Care Service Utilization in the Post-Conflict Democratic Republic of Congo: An Analysis of Health Inequalities over Time
Source: Healthcare (Basel). 2023 Oct 31;11(21):2871. doi: 10.3390/healthcare11212871 (PMC10649172; doi:10.3390/healthcare11212871)
Supplement: Supplementary file 1 [file healthcare-11-02871-s001.zip › Additional file S2 Description of selected variables.pdf]

Additional file S2. Description of selected variables

Dependent Variables	***Description of variables***	***Source***
C-section	Percentage of live births delivered by cesarean section	STAT compiler (DHS)
Prenatal care	The proportion of women aged 15-49 who have attended at least four antenatal care visits for their most recent pregnancy in the five years before the survey (= Last birth during the past 5 years)	STAT compiler (DHS)
Delivery care	The proportion of women aged 15-49 for whom the most recent birth in the five years before the survey was delivered in a health facility. Health facilities could be government, private, NGO (non-governmental organization), or another type such as a maternity clinic	STAT compiler (DHS)
Postnatal care	Last birth during the past 5 years	STAT compiler (DHS)
Independent Variables	***Description of variables***	***Source***
Survey Period		
2007	First survey wave – 2007	STAT compiler (DHS)
2013/2014	Second survey wave – 2013/2014	STAT compiler (DHS)
Geographic Regions		
Eastern Congo	Is centered on the North and South Kivu Provinces, as well as nearby Orientale, Maniema, and Katanga	STAT compiler (DHS)
Western Congo	Includes the capital city (Kinshasa) and the provinces of Bandundu, Bas-Congo, Equateur, Kasaï Oriental, and Kasaï Occidental	STAT compiler (DHS)
Control Variables	***Description of variables***	***Source***
Place of residence		
Urban	Share of population living in urban areas, as defined by national statistical offices	World Development Indicators (World Bank)

Rural	Share of population living in rural areas, as defined by national statistical offices	World Development Indicators (World Bank)
Highest education level	Gross secondary school enrollment as a share of the secondary school-aged population	World Development Indicators (World Bank)
Religion	Religious identification was based on the answer to a single question “What is your religion?”. Various categories of answers were pre-coded in the interview questionnaire based on local knowledge:<br>1 = Catholic; 2 = Protestant; 3 = Salvation army; 4 = Kimbanguist; 5 = other Christian; 6 = Muslim; 7 = Animist; 8 = no religion; 96 = other	STAT compiler (DHS)
Ethnicity	Ethnicity was dichotomized into 1 = Bakongo north and south; 2 = Bas-Kasai and Kwilu-kwango; 3 = Cuvette centrale; 4 = Ubangi and Itimbiri; 5 = Uele lake Albert; 6 = Basele-kivu, Maniema and Kivu; 7 = Kasai, Katanga, Tanganika; 8 = Lunda; 9 = Pygmy; 96 = others	STAT compiler (DHS)
Wealth index	The difference in prevalence for each indicator between the richest (or highest) wealth quintile (q5) and poorest (or lowest) wealth quintile (q1).	STAT compiler (DHS)
Respondent’s current work status	Percent distribution of women and men aged 15-49 employed in the 12 months preceding the survey by occupation (= Percent distribution of women and men by employment status)	STAT compiler (DHS)

*The STAT compiler is a tool that allows users to build custom tables, charts, and maps from thousands of indicators across 90 countries. It is meant to help users compare DHS data across countries and across time.
