# Supplementary material for: Maternal Health Care Service Utilization in the Post-Conflict Democratic Republic of Congo: An Analysis of Health Inequalities over Time
Source: Healthcare (Basel). 2023 Oct 31;11(21):2871. doi: 10.3390/healthcare11212871 (PMC10649172; doi:10.3390/healthcare11212871)

Additional file S3 Lorenz Curves for all selected maternal health care services utilization variables

Ever birth by C-section

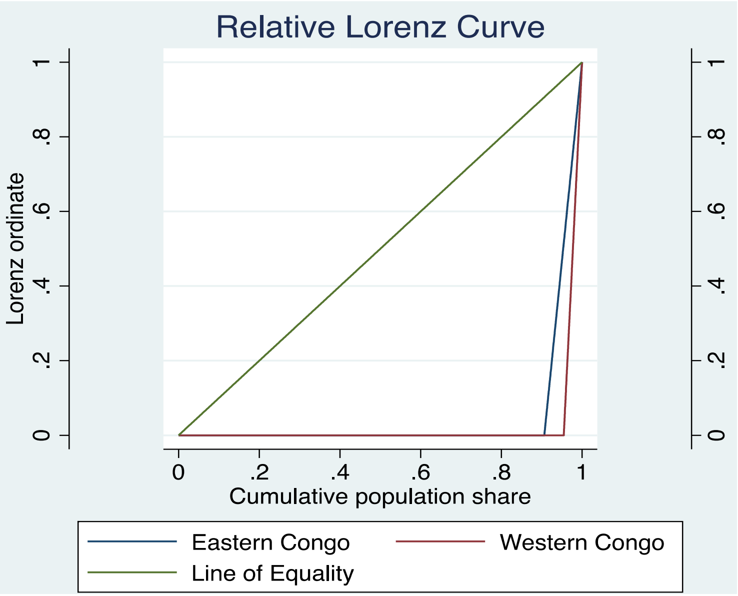

Last birth C-section

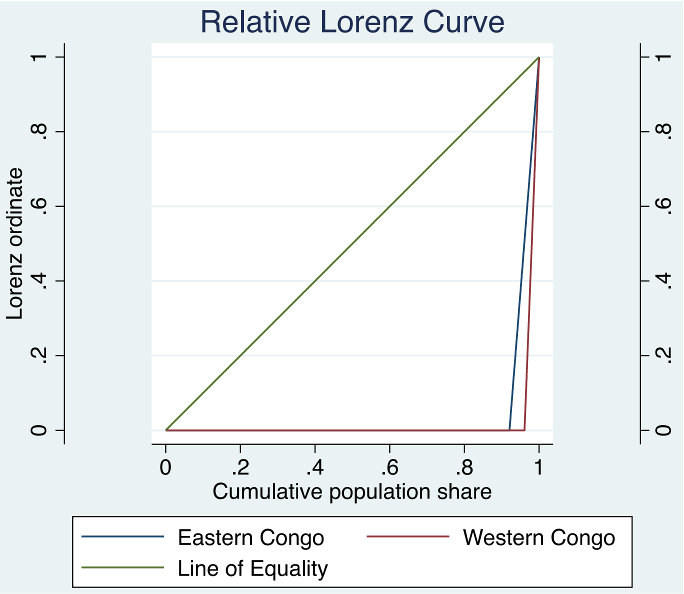

Prenatal Check Number

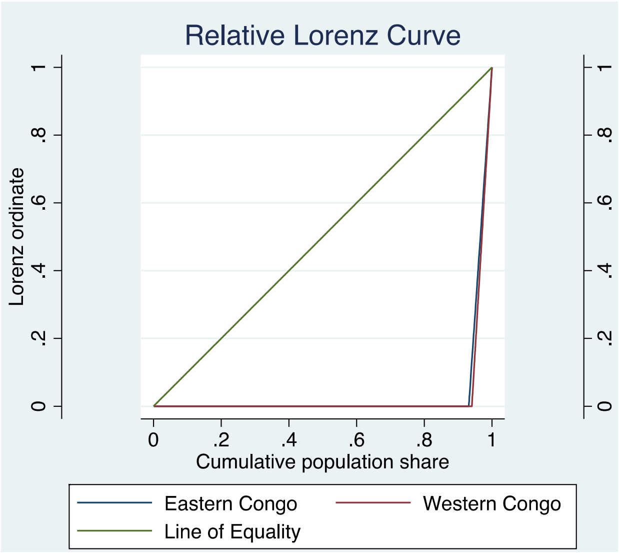

Prenatal check weighed

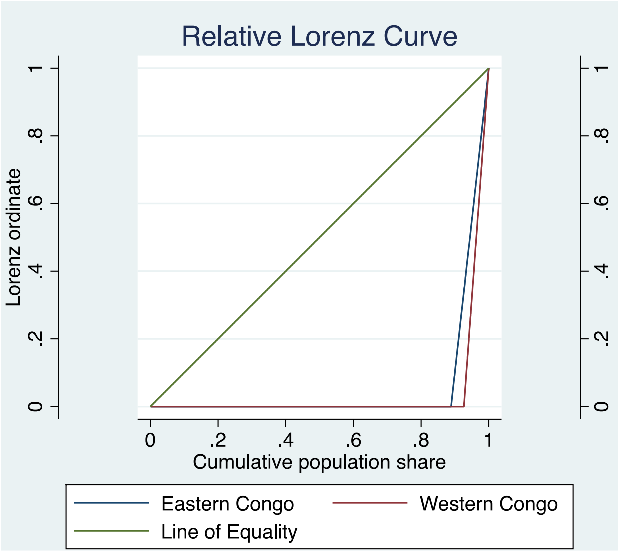

Prenatal check height

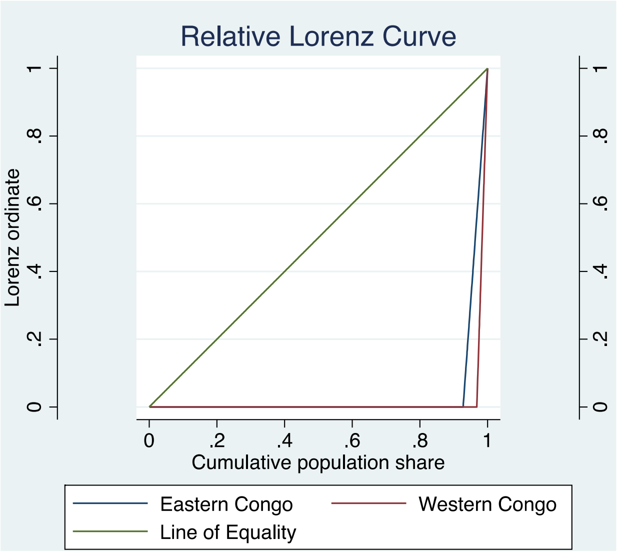

Prenatal check of blood pressure

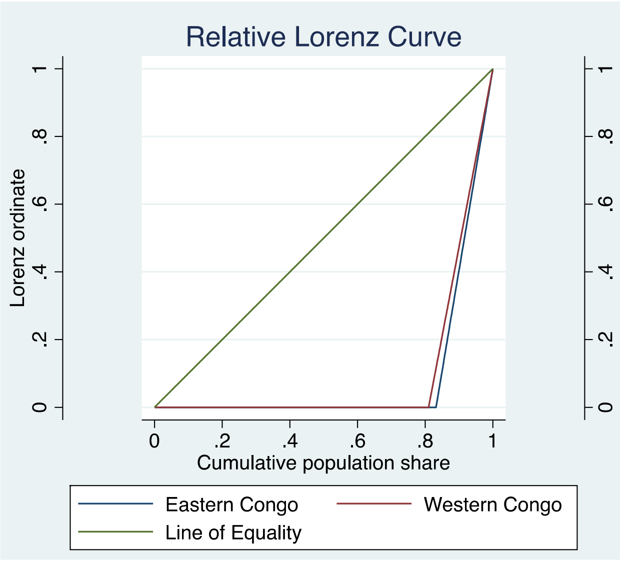

Prenatal check urine sample

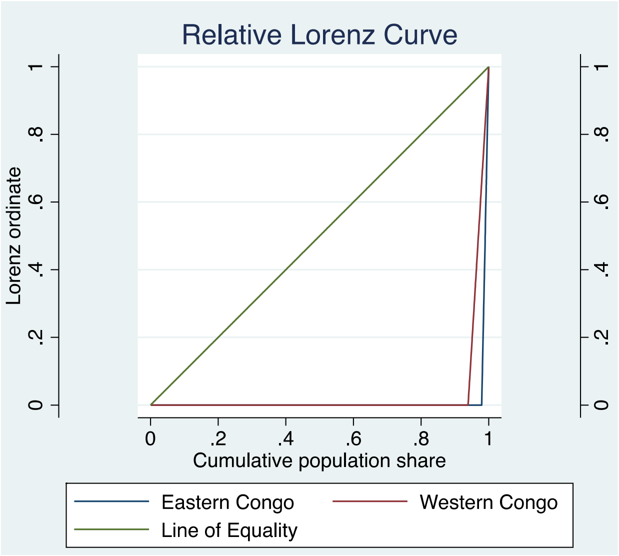

Prenatal check blood sample

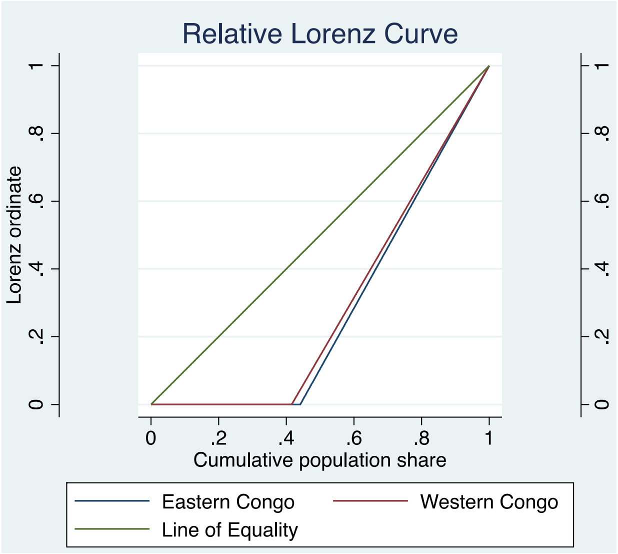

Tetanus injections

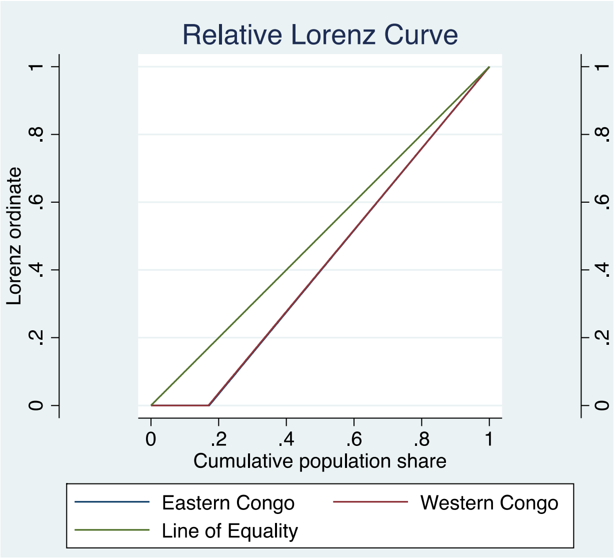

Received pregnancy information

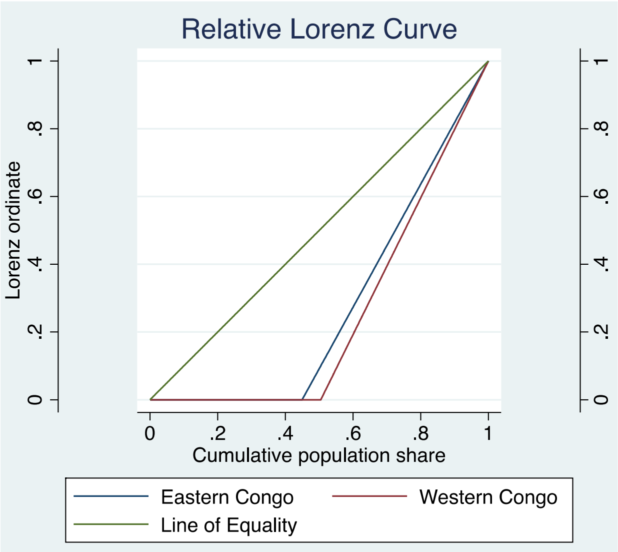

Received postnatal checkup

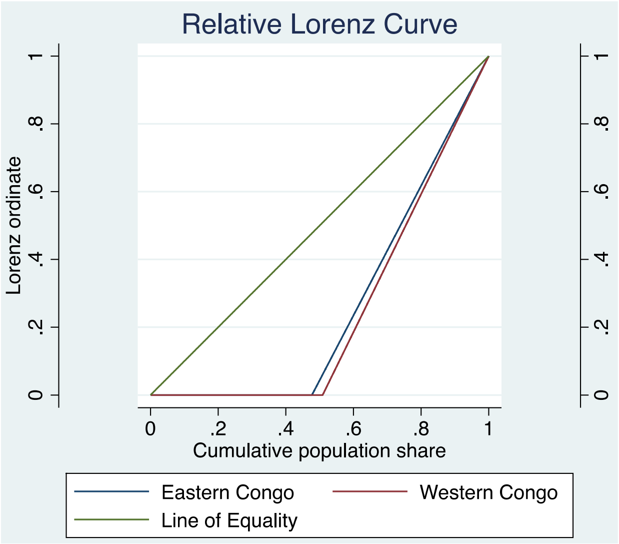

Visited health facilities last 12 months

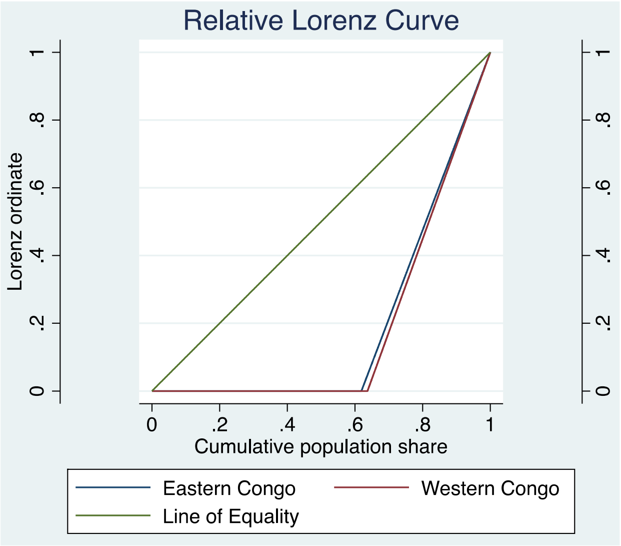

Number of antenatal visits

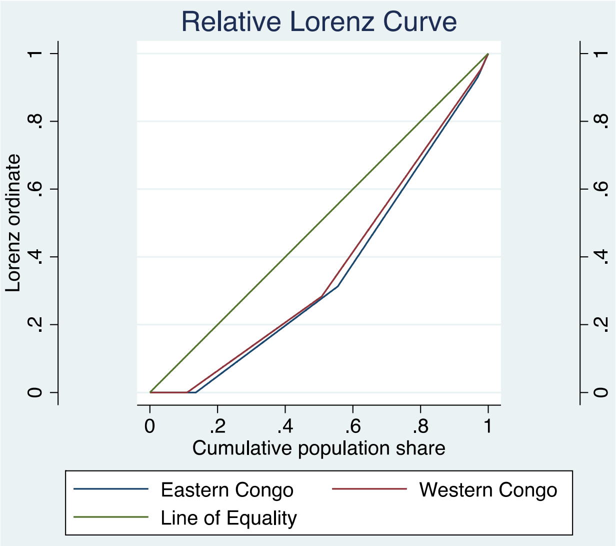

Prenatal care received

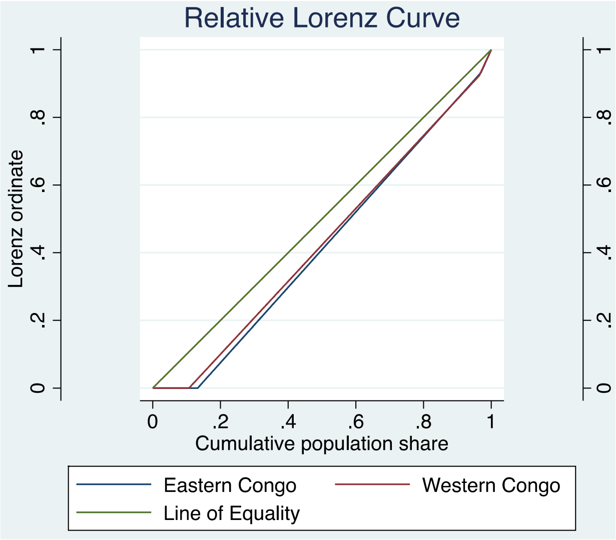

Assistance during delivery

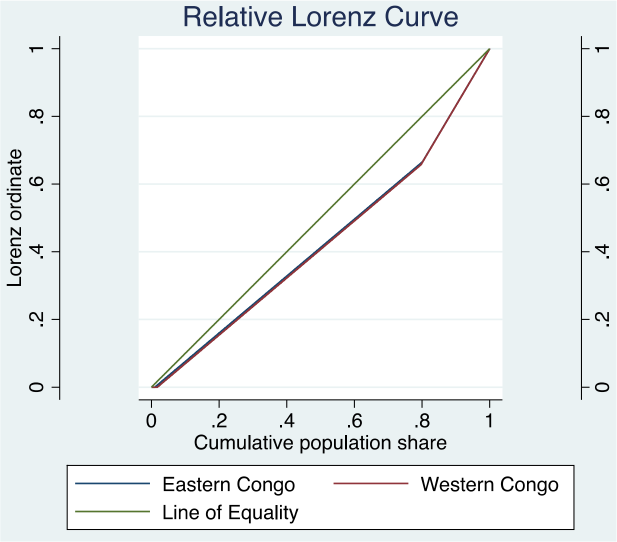

Supplement: Supplementary file 1 [file healthcare-11-02871-s001.zip › Additional file S3 Lorenz Curves for all selected.pdf]
