# Supplementary material for: Maternal Health Care Service Utilization in the Post-Conflict Democratic Republic of Congo: An Analysis of Health Inequalities over Time
Source: Healthcare (Basel). 2023 Oct 31;11(21):2871. doi: 10.3390/healthcare11212871 (PMC10649172; doi:10.3390/healthcare11212871)
Supplement: Supplementary file 1 [file healthcare-11-02871-s001.zip › Table S1 Descriptive characteristics of all respondents in the DRC by survey year.pdf]

Table S1. Descriptive characteristics of all respondents in the DRC by selected variable

Variables	Overall	2007 DHS (N=14.752)
	(%)	(%)
**Place of residence**		
Urban	40.3	47.91
Rural	59.7	52.09
**Highest Education level**		
No education	18.96	21.08
Primary	38.51	37.8
Secondary	39.58	38.21
Higher	2.96	2.91
**Region**		
Kinshasa	12.04	16.67
Bandundu	11.85	9.42
Bas-congo	5.81	7.3
Equateur	12.5	9.07
Kasai Occidental	7.59	7.27
Kasai Oriental	10.2	8.66
Katanga	10.83	9.25
Maniema	5.93	8.54
Nord-Kivu	6.84	8.16
Orientale	10.03	7.55
Sud-Kivu	6.38	8.07
**Wealth Index**		
Poorest	21.75	19.03
Poorer	19.09	17.64
Middle	19.05	18.38
Richer	18.76	20.17
Richest	21.35	24.78
**Religion**		
Catholic	29.14	29.67
Protestant	28.84	30.7
Salvation army	0.24	0.36
Kimbanguist	3.07	3.23
Other christian	34.74	32.33
Muslim	1.69	1.96
Animist	0.42	0.52
No religion	0.92	1.02
Bundu dia kongo	0.08	
Vuvamu	0.02	
Other	0.65	0.13
.	0.19	0.08
**Ethnicity**		
Bakongo north and south	10.29	13.69
Bas-kasai and kwilu-kwango	14.86	12.94

Cuvette centrale	9.1	8.23
Ubangi and itimbiri	9.81	6.29
Uele lake albert	7.34	4.85
Basele-k, man. and kivu	19.75	25.12
Kasai, katanga, tanganika	26.88	26.76
Lunda	1.01	0.98
Pygmy	0.22	0.09
Foreign/Non-congolese	0.32	
Others	0.25	0.71
.	0.16	0.33
**Currently working**		
No	33.76	38
Yes	65.99	61.93
.	0.25	0.07
**Marital Status**		
Never married	24.36	24.76
Married	51.54	56.03
Living together	14.5	9.86
Widowed	2.26	2.11
Divorced	1.94	1.72
Not living together	5.4	5.51

Data are presented as N and percentage

les and by survey wave

2013-14 DHS (N=27.483)
(%)
36.26
63.74
17.83
38.88
40.31
2.98
9.58
13.14
5.02
14.32
7.76
11.01
11.66
4.54
6.13
11.35
5.49
23.19
19.87
19.41
18.01
19.53
28.86
27.85
0.18
2.99
36.02
1.54
0.37
0.87
0.12
0.03
0.93
0.25
8.49
15.88

9.56
11.68
8.66
16.9
26.95
1.03
0.29
0.49
0.01
0.07
31.51
68.15
0.35
24.14
49.15
16.96
2.34
2.06
5.34
