# Supplementary material for: Maternal Health Care Service Utilization in the Post-Conflict Democratic Republic of Congo: An Analysis of Health Inequalities over Time
Source: Healthcare (Basel). 2023 Oct 31;11(21):2871. doi: 10.3390/healthcare11212871 (PMC10649172; doi:10.3390/healthcare11212871)
Supplement: Supplementary file 1 [file healthcare-11-02871-s001.zip › Table S3 Relative Risk Ratio on categorical variables - Multinomial regressions.pdf]

Table S3. Relative Risk Ratio on categorical variables - Mul							
**Variables**		**Number Antenatal visits**				**Prenatalcare received from**	
	**No visits**	**1-3 visits**	**4-7 visits**	**8 or more visits**	**Base = no one**	**Professional care**	**Traditional care**
	Relative Risk Ratio	*Relative Risk Ratio*	*Relative Risk Ratio*	*Relative Risk Ratio*	*Relative Risk Ratio*	*Relative Risk Ratio*	*Relative Risk Ratio*
Base = Eastern Congo							
**Western Congo**		1.577	2.372***	1290		1.746**	5.205***
		(0.953 - 2.609)	(1.406 - 4.002)	(0.381 - 4.372)		(1.099 - 2.775)	(2.124 - 12.76)
Base = 2013-2014							
**2007**		0.734	0.711	1,010		0.784	0.565
		(0.497 - 1.082)	(0.475 - 1.064)	(0.427 - 2.389)		(0.543 - 1.133)	(0.257 - 1.240)
Base = Catholic							
**Protestant**		0.919	0.926	0.422		0.839	1,156
		(0.566 - 1.494)	(0.565 - 1.518)	(0.139 - 1.280)		(0.525 - 1.341)	(0.432 - 3.092)
**Kimbanguist**		0.707	0.437	0.185		0.519	0.427
		(0.304 - 1.647)	(0.181 - 1.055)	(0.0246 - 1.387)		(0.233 - 1.156)	(0.0809 - 2.254)
**Other Christians**		0.876	0.882	0.604		0.870	0.662
		(0.544 - 1.410)	(0.543 - 1.432)	(0.209 - 1.747)		(0.551 - 1.373)	(0.256 - 1.710)
**Muslim**		0.396	0.454	0***		0.290**	4,015
		(0.107 - 1.457)	(0.115 - 1.794)	(0 - 0)		(0.0859 - 0.979)	(0.612 - 26.34)
**Animist**		0.347	1,441	0***		0.714	0.730
		(0.0394 - 3.060)	(0.111 - 18.79)	(0 - 0)		(0.0851 - 5.982)	(0.0373 - 14.27)
**No religion**		0.240**	0.263**	0.0250***		0.233***	0***
		(0.0779 - 0.737)	(0.0841 - 0.824)	(0.00236 - 0.265)		(0.0894 - 0.607)	(0 - 0)
**Other**		0.700	0.158***	0***		0.348**	1,477
		(0.265 - 1.850)	(0.0422 - 0.592)	(0 - 0)		(0.132 - 0.918)	(0.284 - 7.694)
Base = Bakongo north and South							
**Bas-Kasai and Kwilu-Kwngo**		0.916	1097	0.813		1,034	3,614
		(0.335 - 2.504)	(0.395 - 3.047)	(0.0694 - 9.514)		(0.385 - 2.772)	(0.552 - 23.68)
**Cuvette Central**		0.313**	0.819	9.33		0.568	3,118
		(0.116 - 0.847)	(0.301 - 2.228)	(0.862 - 100.9)		(0.222 - 1.455)	(0.519 - 18.72)
**Ubangi and Itimbiri**		0.553	0.789	4,036		0.668	1,078
		(0.204 - 1.495)	(0.287 - 2.169)	(0.375 - 43.48)		(0.256 - 1.741)	(0.133 - 8.756)
**Uele Lake Albert**		0.861	1,631	3,714		1,083	17.07**
		(0.258 - 2.873)	(0.462 - 5.750)	(0.245 - 56.40)		(0.342 - 3.431)	(1.890 - 154.1)
**Basele-k, man. And Kivu**		2,387	4.415**	8997		3.028**	13.41**
		(0.775 - 7.352)	(1.378 - 14.15)	(0.650 - 124.5)		(1.024 - 8.952)	(1.595 - 112.7)
**Kasai, Katanga, Tanganika**		0.483	0.618	4430		0.560	1,912
		(0.194 - 1.202)	(0.243 - 1.574)	(0.475 - 41.29)		(0.231 - 1.354)	(0.319 - 11.44)
**Lunda**		1,000	2222	33.47**		1,602	0***
		(0.210 - 4.757)	(0.473 - 10.43)	(1.741 - 643.4)		(0.382 - 6.718)	(0 - 1.07e-10)
**Other**		0.531	0.741	0***		0.572	4,955
		(0.0966 - 2.921)	(0.114 - 4.812)	(0 - 0)		(0.110 - 2.984)	(0.247 - 99.41)
Base = Urban							
**Rural**		0.719	0.690	3.636**		0.726	2.576
		(0.404 - 1.281)	(0.390 - 1.222)	(1.207 - 10.95)		(0.425 - 1.242)	(0.976 - 6.798)
Base = Poorest							
**Poorer**		0.941	1300	1851		1,169	0.769
		(0.608 - 1.457)	(0.829 - 2.037)	(0.646 - 5.303)		(0.777 - 1.760)	(0.340 - 1.741)
**Middle**		1.699**	1.928**	2317		1.959***	0.387
		(1.046 - 2.761)	(1.158 - 3.210)	(0.747 - 7.193)		(1.231 - 3.116)	(0.144 - 1.040)
**Richer**		1342	1.928**	0.825		1.585	1,365
		(0.753 - 2.395)	(1.075 - 3.458)	(0.142 - 4.795)		(0.932 - 2.695)	(0.509 - 3.657)
**Richest**		0.684	1970	7.077**		1,257	2,451
		(0.233 - 2.010)	(0.683 - 5.682)	(1.225 - 40.90)		(0.455 - 3.478)	(0.492 - 12.22)
Base = no - currently not working							
**Yes - currently working**		1029	1129	0.571		1,060	1,749
		(0.677 - 1.565)	(0.747 - 1.705)	(0.221 - 1.477)		(0.719 - 1.562)	(0.802 - 3.814)
Base = no education							
**Primary education level**		1.481	1.752***	1594		1.512**	3.554***
		(0.995 - 2.205)	(1.149 - 2.671)	(0.604 - 4.209)		(1.037 - 2.204)	(1.567 - 8.061)
**Secondary education level**		3.285***	5.633***	3.828**		4.497***	4.643***
		(1.769 - 6.100)	(3.039 - 10.44)	(1.152 - 12.72)		(2.476 - 8.165)	(1.647 - 13.09)
**Higher education level**		2674	3784	32.45**		3,646	0***
		(0.234 - 30.59)	(0.416 - 34.44)	(1.435 - 733.6)		(0.411 - 32.35)	(0 - 0)
**Constant**		3.441*	1081	0.0111***		4.264**	0.00446***
		(0.919 - 12.88)	(0.277 - 4.222)	(0.000571 - 0.215)		(1.228 - 14.81)	(0.000293 - 0.0677)
**Observations**	2.537	2.537	2.537	2.537	2.568	2.568	2.568

Notes_Titles

Relative risk measures the association between the exposure and the outcome.
Robust ci in parentheses (Figures in brackets show 95 percent confidence intervals).
**** p<0.01, ** p<0.05*
