# Supplementary material for: Maternal Health Care Service Utilization in the Post-Conflict Democratic Republic of Congo: An Analysis of Health Inequalities over Time
Source: Healthcare (Basel). 2023 Oct 31;11(21):2871. doi: 10.3390/healthcare11212871 (PMC10649172; doi:10.3390/healthcare11212871)
Supplement: Supplementary file 1 [file healthcare-11-02871-s001.zip › Table S4 Gini coefficients for all selected maternal health variables.pdf]

Table S4. Gini coefficients for all selected maternal health variables						
	Eastern DRC			Western DRC		
**All selected Variables**	**Overall**	**2007**	**2013/14**	**Overall**	**2007**	**2013/14**
**Cesarean-section**						
Ever birth C-section	0.91	0.94	0.90	0.96	0.96	0.96
Last birth C-section	0.93	0.95	0.92	0.97	0.96	0.97
**Prenatal care**						
Prenatal check_no	0.94	0.93	0.94	0.95	0.95	0.95
Received prenatal care	0.17	0.24	0.13	0.15	0.14	0.15
Prenatal check weighed	0.89	0.88	0.88	0.93	0.92	0.94
Prenatal check height	0.93	0.97	0.91	0.97	0.97	0.98
Prenatal check blood pressure	0.84	0.83	0.84	0.82	0.82	0.81
Prenatal check urine sample	0.98	0.97	0.99	0.94	0.95	0.94
Prenatal check blood sample	0.45	0.45	0.45	0.42	0.42	0.42
Tetanus injections	0.18	0.24	0.15	0.17	0.19	0.17
Received pregnancy information	0.45	0.61	0.39	0.51	0.67	0.43
**Postnatal Care**						
Received postnatal checkup	0.48	0.04	0.49	0.51	0.13	0.52
Visited health facilities last 12 months	0.62	0.66	0.61	0.64	0.67	0.62
Assistance during delivery	0.15	0.17	0.14	0.16	0.17	0.15
