# Supplementary material for: Maternal Health Care Service Utilization in the Post-Conflict Democratic Republic of Congo: An Analysis of Health Inequalities over Time
Source: Healthcare (Basel). 2023 Oct 31;11(21):2871. doi: 10.3390/healthcare11212871 (PMC10649172; doi:10.3390/healthcare11212871)
Supplement: Supplementary file 1 [file healthcare-11-02871-s001.zip › Table S5 Concentration indices by maternal health variables and by survey year 2007 and 2013-2014.pdf]

Table S5. Concentration indices by maternal health variables, by Survey year 2007 and 2013-2014, and by regions Western-Eastern Congo																
	2007				2013/14				Western Congo				Eastern Congo			
All selected Variables	Group 0 = Eastern Congo	Group 1 = Western Congo	Socioeconomic inequality in the health variable	Statistically significance between the 2 groups in the socioeconomic inequality	Group 0 = Eastern Congo	Group 1 = Western Congo	Socioeconomic inequality in the health variable	Statistically significance between the 2 groups in the socioeconomic inequality	CI	PeriodSurvey = 0 (2013/2014)	PeriodSurvey =1 (2007)	Test for Stat. Significant Differences	CI	PeriodSurvey =0 (2013/2014)	PeriodSurvey =1 (2007)	Test for Stat. Significant Differences
**Delivery**																
Ever C-section	0.67	0.68	0.68	0.97	0.65	0.68	0.67	0.89	0.68	0.68	0.68	0.97	0.65	0.65	0.67	0.97
Last birth C-section	0.68	0.69	0.68	1.00	0.66	0.69	0.68	0.82	0.69	0.69	0.69	0.94	0.66	0.66	0.68	0.87
**Prenatal care**																
Prenatal care received	0.47	0.85	0.63	0.47	0.53	0.67	0.59	0.75	0.73	0.67	0.85	0.80	0.52	0.53	0.47	0.83
Prenatal check number	0.67	0.68	0.68	0.97	0.68	0.68	0.68	0.92	0.68	0.68	0.68	0.87	0.67	0.68	0.67	0.91
Prenatal check weighed	0.64	0.67	0.66	0.90	0.65	0.67	0.67	0.82	0.67	0.67	0.67	0.88	0.65	0.65	0.64	0.95
Prenatal check height	0.69	0.69	0.69	0.98	0.66	0.69	0.68	0.54	0.69	0.69	0.69	0.93	0.67	0.66	0.69	0.55
Prenatal check blood pressure	0.59	0.59	0.59	0.71	0.61	0.58	0.59	0.90	0.58	0.58	0.59	0.86	0.60	0.61	0.59	0.74
Prenatal check urine sample	0.69	0.68	0.69	0.53	0.70	0.67	0.68	0.46	0.68	0.67	0.68	0.67	0.70	0.70	0.69	0.91
Prenatal check bloodsample	0.22	0.29	0.26	0.44	0.21	0.24	0.23	0.58	0.26	0.24	0.29	0.79	0.21	0.21	0.22	0.27
Tetanus injections	0.53	0.58	0.56	0.88	0.62	0.59	0.60	0.74	0.59	0.59	0.58	0.93	0.60	0.62	0.53	0.67
Received pregnancy information	0.29	0.31	0.31	0.02	0.26	0.21	0.23	0.71	0.03	0.21	0.31	0.24	0.10	0.26	0.29	0.01
Number antenatal visits	0.49	0.11	0.31	0.91	0.13	0.12	0.12	0.72	0.11	0.12	0.11	0.63	0.23	0.13	0.49	0.75
**Postnatal care**																
Received postnatal checkup	0.70	0.65	0.66	0.49	0.09	0.02	0.03	0.28	0.01	0.02	0.65	0.00	0.10	0.09	0.70	0.00
Visited health facilities last 12 months	0.37	0.38	0.37	0.93	0.25	0.30	0.28	0.37	0.33	0.30	0.38	0.71	0.29	0.25	0.37	0.83
Assistance during delivery	0.40	0.49	0.46	0.88	0.49	0.46	0.47	0.88	0.47	0.46	0.49	0.97	0.46	0.49	0.40	0.78
