# Supplementary material for: Maternal Health Care Service Utilization in the Post-Conflict Democratic Republic of Congo: An Analysis of Health Inequalities over Time
Source: Healthcare (Basel). 2023 Oct 31;11(21):2871. doi: 10.3390/healthcare11212871 (PMC10649172; doi:10.3390/healthcare11212871)
Supplement: Supplementary file 1 [file Table S2 Odds ratio by socio-demographic characteristics-Logistic regression.pdf › Table S2 Odds ratio by socio-demographic characteristics-Logistic regression.pdf]

Table S2. Odds ratio of all selected maternal health variables by socio-demogra												
	Delivery						Antenatal care					Postnatal Care
Variables	EverbirthCsection	LastbirthCsection	Number	Weighed	Height	Blood pressure	Urine sample	Blood sample	Tetainjectbp2	Receivedinforegcompl	Receivedpostnatcheckup	Visitedhealthfaclast12months
	odds ratio	*odds ratio*	*odds ratio*	*odds ratio*	*odds ratio*	*odds ratio*	*odds ratio*	*odds ratio*	*odds ratio*	*odds ratio*	*odds ratio*	*odds ratio*
Base = Eastern Congo												
**Western Congo**	1,025	1,236	1,444	1,277	0.298***	1476	3.745***	0.623**	0.841	0.751	0.997	1,009
	(0.707 - 1.487)	(0.886 - 1.725)	(0.715 - 2.916)	(0.647 - 2.521)	(0.147 - 0.607)	(0.780 - 2.794)	(1.593 - 8.805)	(0.400 - 0.971)	(0.509 - 1.387)	(0.501 - 1.126)	(0.837 - 1.187)	(0.905 - 1.126)
Base = 2013-2014												
**2007**	0.793**	0.823	0.921	1,252	0.794	1,061	1,006	0.949	0.758	0.373***	13.06***	0.836***
	(0.655 - 0.959)	(0.676 - 1.001)	(0.527 - 1.609)	(0.787 - 1.992)	(0.393 - 1.604)	(0.737 - 1.527)	(0.566 - 1.785)	(0.713 - 1.262)	(0.539 - 1.066)	(0.282 - 0.493)	(7.255 - 23.51)	(0.777 - 0.899)
Base = Catholic												
**Protestant**	0.920	0.819	1005	0.792	1163	0.873	0.705	1270	1035	1201	1032	1046
	(0.720 - 1.176)	(0.632 - 1.060)	(0.517 - 1.952)	(0.461 - 1.359)	(0.594 - 2.280)	(0.561 - 1.359)	(0.361 - 1.375)	(0.900 - 1.793)	(0.695 - 1.541)	(0.855 - 1.686)	(0.890 - 1.197)	(0.953 - 1.148)
**Kimbanguist**	0.404***	0.357***	1745	0.204**	0.145	0.877	0.527	1634	0.820	0.952	0.484***	0.811
	(0.221 - 0.740)	(0.184 - 0.693)	(0.511 - 5.959)	(0.0466 - 0.895)	(0.0181 - 1.162)	(0.381 - 2.018)	(0.0634 - 4.376)	(0.717 - 3.725)	(0.353 - 1.905)	(0.485 - 1.871)	(0.338 - 0.693)	(0.653 - 1.008)
**Other Christians**	0.781**	0.743**	1269	0.896	1663	0.806	0.572	1173	1221	0.829	0.783***	1033
	(0.619 - 0.984)	(0.578 - 0.955)	(0.604 - 2.666)	(0.507 - 1.584)	(0.831 - 3.327)	(0.519 - 1.252)	(0.295 - 1.107)	(0.826 - 1.666)	(0.802 - 1.860)	(0.591 - 1.164)	(0.676 - 0.906)	(0.945 - 1.130)
**Muslim**	0.544	0.543	2.753	1570	0.187	0.254	1163	1264	3033	0.600	0.840	0.907
	(0.287 - 1.033)	(0.274 - 1.074)	(0.882 - 8.601)	(0.550 - 4.482)	(0.0194 - 1.802)	(0.0350 - 1.837)	(0.138 - 9.828)	(0.467 - 3.426)	(0.698 - 13.18)	(0.159 - 2.259)	(0.527 - 1.340)	(0.692 - 1.190)
**Animist**	0.529	0.574	4291			1015		1419	1603	2043	0.926	1421
	(0.0861 - 3.254)	(0.0938 - 3.512)	(0.646 - 28.50)			(0.190 - 5.428)		(0.396 - 5.081)	(0.435 - 5.904)	(0.533 - 7.832)	(0.438 - 1.956)	(0.888 - 2.272)
**No religion**	0.517	0.462		0.254		0.567	2172	3.945**	1554	1215	0.802	1024
	(0.167 - 1.601)	(0.118 - 1.810)		(0.0481 - 1.339)		(0.109 - 2.949)	(0.479 - 9.852)	(1.146 - 13.58)	(0.380 - 6.350)	(0.418 - 3.530)	(0.485 - 1.326)	(0.692 - 1.513)
**Other**	1137	1124	1571	3.293	2679	0.889		0.341	0.661	1154	0.551***	0.960
	(0.472 - 2.741)	(0.436 - 2.900)	(0.291 - 8.483)	(0.824 - 13.16)	(0.719 - 9.983)	(0.183 - 4.324)		(0.0820 - 1.416)	(0.185 - 2.356)	(0.401 - 3.319)	(0.357 - 0.852)	(0.697 - 1.321)
Base = Bakongo north and South												
**Bas-Kasai and Kwilu-Kwngo**	0.595***	0.493***	8.754	7.987***	7.777**	0.641	3430	0.475**	0.425**	0.650	1.267**	0.985
	(0.410 - 0.863)	(0.341 - 0.713)	(0.879 - 87.13)	(1.879 - 33.95)	(1.315 - 45.98)	(0.307 - 1.340)	(0.766 - 15.37)	(0.259 - 0.870)	(0.187 - 0.966)	(0.380 - 1.111)	(1.001 - 1.604)	(0.866 - 1.122)
**Cuvette Central**	0.560**	0.463***	13.85**	5.978**	1653	1284	2920	0.380***	0.593	0.951	0.582***	1.321***
	(0.335 - 0.936)	(0.265 - 0.809)	(1.559 - 123.1)	(1.146 - 31.19)	(0.244 - 11.22)	(0.565 - 2.920)	(0.560 - 15.22)	(0.188 - 0.767)	(0.250 - 1.407)	(0.477 - 1.893)	(0.440 - 0.771)	(1.128 - 1.548)
**Ubangi and Itimbiri**	0.535***	0.435***	6162	1044	2512	0.981	11.64***	0.537	0.840	0.631	0.435***	1.335***
	(0.362 - 0.791)	(0.285 - 0.665)	(0.680 - 55.83)	(0.222 - 4.909)	(0.417 - 15.14)	(0.465 - 2.068)	(2.791 - 48.53)	(0.285 - 1.014)	(0.355 - 1.991)	(0.351 - 1.135)	(0.338 - 0.560)	(1.158 - 1.541)
**Uele Lake Albert**	1360	1434	34.34***	21.52***	1027	1389	0.401	0.167***	0.487	0.395**	0.806	1022
	(0.806 - 2.294)	(0.855 - 2.404)	(3.445 - 342.3)	(4.270 - 108.5)	(0.127 - 8.335)	(0.481 - 4.013)	(0.0333 - 4.820)	(0.0752 - 0.369)	(0.174 - 1.367)	(0.187 - 0.834)	(0.590 - 1.101)	(0.842 - 1.240)
**Basele-k, man. And Kivu**	2.111***	2.372***	16.19**	9.505***	4289	1286	7.827**	0.215***	0.490	0.675	1143	1.433***
	(1.322 - 3.371)	(1.548 - 3.636)	(1.623 - 161.6)	(1.968 - 45.90)	(0.694 - 26.51)	(0.479 - 3.455)	(1.463 - 41.88)	(0.103 - 0.449)	(0.193 - 1.246)	(0.342 - 1.329)	(0.854 - 1.531)	(1.215 - 1.690)
**Kasai, Katanga, Tanganika**	0.630***	0.611***	23.73***	7.352***	3202	1463	6.586***	0.219***	0.504	0.515**	0.675***	1.235***
	(0.447 - 0.888)	(0.432 - 0.863)	(2.773 - 203.0)	(1.781 - 30.36)	(0.557 - 18.42)	(0.739 - 2.895)	(1.577 - 27.51)	(0.123 - 0.393)	(0.229 - 1.107)	(0.308 - 0.859)	(0.536 - 0.849)	(1.096 - 1.393)
**Lunda**	1486	1618	4774		0.600	0.375		1265	0.768	0.462	1102	1.712***
	(0.649 - 3.400)	(0.693 - 3.777)	(0.239 - 95.49)	(0.755 - 33.42)	(0.0410 - 8.780)	(0.0744 - 1.893)		(0.389 - 4.121)	(0.148 - 3.983)	(0.120 - 1.774)	(0.608 - 1.998)	(1.205 - 2.432)
**Other**	0.458	0.523	39.47***	1703	5772		41.64***	0.214	1728	0.491	0.705	1083
	(0.166 - 1.266)	(0.189 - 1.444)	(2.957 - 526.8)	(0.124 - 23.40)	(0.375 - 88.82)		(4.806 - 360.8)	(0.0407 - 1.124)	(0.178 - 16.74)	(0.133 - 1.816)	(0.371 - 1.342)	(0.737 - 1.592)
Base = Urban												
**Rural**	0.788	0.755**	1232	1088	1478	1.897***	0.717	0.631**	0.541**	0.721	0.641***	0.884**
	(0.610 - 1.018)	(0.579 - 0.985)	(0.569 - 2.668)	(0.551 - 2.147)	(0.706 - 3.091)	(1.173 - 3.068)	(0.337 - 1.524)	(0.440 - 0.904)	(0.333 - 0.880)	(0.500 - 1.038)	(0.547 - 0.751)	(0.794 - 0.984)
Base = Poorest												
**Poorer**	1013	1156	0.678	0.885	0.752	0.910	0.938	1.407	1.547**	1078	1112	1.130**
	(0.729 - 1.407)	(0.813 - 1.644)	(0.375 - 1.224)	(0.517 - 1.514)	(0.351 - 1.609)	(0.575 - 1.438)	(0.433 - 2.032)	(0.959 - 2.064)	(1.007 - 2.377)	(0.751 - 1.549)	(0.947 - 1.306)	(1.011 - 1.264)
**Middle**	1170	1301	0.823	0.988	0.420**	0.885	1144	1.407	1.691**	1220	1.236**	1090
	(0.838 - 1.634)	(0.913 - 1.854)	(0.464 - 1.459)	(0.554 - 1.762)	(0.194 - 0.911)	(0.555 - 1.413)	(0.558 - 2.346)	(0.972 - 2.037)	(1.086 - 2.632)	(0.846 - 1.759)	(1.049 - 1.457)	(0.973 - 1.221)
**Richer**	1.755***	1.726***	0.298**	0.468	0.965	0.599	0.601	2.710***	1325	1372	1.401***	1.125
	(1.244 - 2.477)	(1.217 - 2.447)	(0.112 - 0.790)	(0.216 - 1.011)	(0.432 - 2.156)	(0.350 - 1.026)	(0.224 - 1.615)	(1.753 - 4.189)	(0.794 - 2.213)	(0.887 - 2.122)	(1.156 - 1.697)	(0.988 - 1.281)
**Richest**	1.711***	1.757***	0.105**	0.143**	0.436	0.394**	0.709	4.180***	1294	1218	1.824***	1126
	(1.173 - 2.497)	(1.183 - 2.610)	(0.0179 - 0.623)	(0.0247 - 0.825)	(0.107 - 1.772)	(0.166 - 0.935)	(0.213 - 2.361)	(2.254 - 7.749)	(0.613 - 2.731)	(0.709 - 2.091)	(1.444 - 2.304)	(0.971 - 1.306)
Base = no - currently not working												
**Yes - currently working**	0.956	1027	1192	1626	0.612	0.831	0.931	1045	0.789	1007	0.894	1.502***
	(0.769 - 1.189)	(0.826 - 1.277)	(0.672 - 2.114)	(0.909 - 2.908)	(0.341 - 1.101)	(0.564 - 1.225)	(0.498 - 1.741)	(0.761 - 1.436)	(0.538 - 1.158)	(0.756 - 1.341)	(0.786 - 1.017)	(1.393 - 1.621)
Base = no education												
**Primary education level**	0.827	0.799	1070	0.861	1225	0.854	1185	1128	1184	0.827	1.170**	1.202***
	(0.624 - 1.098)	(0.597 - 1.069)	(0.615 - 1.862)	(0.511 - 1.450)	(0.648 - 2.316)	(0.562 - 1.297)	(0.558 - 2.518)	(0.806 - 1.578)	(0.802 - 1.747)	(0.591 - 1.157)	(1.006 - 1.359)	(1.084 - 1.332)
**Secondary education level**	1083	1043	0.410**	0.327***	0.711	0.833	0.834	2.231***	1.702**	1322	1.574***	1.309***
	(0.788 - 1.487)	(0.760 - 1.431)	(0.195 - 0.862)	(0.161 - 0.665)	(0.293 - 1.726)	(0.500 - 1.387)	(0.357 - 1.944)	(1.492 - 3.336)	(1.041 - 2.783)	(0.885 - 1.974)	(1.326 - 1.869)	(1.167 - 1.468)
**Higher education level**	1.669	1.860**				0.0750**		59.56***	4726	23.93***	3.805***	1.584***
	(0.937 - 2.973)	(1.033 - 3.352)				(0.00841 - 0.669)		(6.627 - 535.2)	(0.514 - 43.43)	(3.161 - 181.2)	(2.247 - 6.443)	(1.284 - 1.955)
**Constant**	0.0823***	0.0633***	0.00429***	0.0150***	0.0353***	0.181***	0.00660***	2.838**	10.85***	2.956**	1,216	0.310***
	(0.0450 - 0.150)	(0.0350 - 0.114)	(0.000318 - 0.0580)	(0.00210 - 0.107)	(0.00401 - 0.311)	(0.0559 - 0.585)	(0.000916 - 0.0475)	(1.120 - 7.189)	(3.345 - 35.17)	(1.219 - 7.168)	(0.834 - 1.773)	(0.247 - 0.389)
**Observations**	16.609	16.592	2.163	2.164	2.146	2.184	2.11	2.198	2.169	2.173	11.37	28.643

Notes_Titles

Robust ci in parentheses (Figures in brackets show 95 percent confidence intervals).

****** p<0.01, ** p<0.05*** (to facilitate readability-only significant results have been presented; please refer to appendix x for the complete results of the analyses)
